# Computational methods for *ab initio* detection of microRNAs

**DOI:** 10.3389/fgene.2012.00209

**Published:** 2012-10-10

**Authors:** Jens Allmer, Malik Yousef

**Affiliations:** ^1^Department of Molecular Biology and Genetics, Izmir Institute of TechnologyUrla, Turkey; ^2^The Galilee Society Institute of Applied ResearchShefa-’Amr, Israel

**Keywords:** mature miRNA, ab initio, prediction of miRNAs, prediction accuracy

## Abstract

MicroRNAs are small RNA sequences of 18–24 nucleotides in length, which serve as templates to drive post-transcriptional gene silencing. The canonical microRNA pathway starts with transcription from DNA and is followed by processing via the microprocessor complex, yielding a hairpin structure. Which is then exported into the cytosol where it is processed by Dicer and then incorporated into the RNA-induced silencing complex. All of these biogenesis steps add to the overall specificity of miRNA production and effect. Unfortunately, their modes of action are just beginning to be elucidated and therefore computational prediction algorithms cannot model the process but are usually forced to employ machine learning approaches. This work focuses on *ab initio* prediction methods throughout; and therefore homology-based miRNA detection methods are not discussed. Current *ab initio* prediction algorithms, their ties to data mining, and their prediction accuracy are detailed.

## INTRODUCTION

MicroRNAs (miRNAs) are a group of small non-coding RNAs, discovered in the early 90s by Ambros and colleagues (Lee et al., 1993), which convey post-transcriptional regulation. In most cases miRNAs lead to down regulation of their target mRNAs but translational activation has been observed (Ørom et al., 2008). It has been estimated that 60% of all human genes are regulated by miRNAs (Friedman et al., 2009). Another estimate is that there are more than 1000 miRNAs in the human genome, (Berezikov et al., 2005) and with currently about 1500 human miRNAs in miRBase (Griffiths-Jones et al., 2008; including passenger and guide strands), this number will likely be surpassed soon. MiRNAs can come from introns (Morlando et al., 2008), coding regions (Rodriguez et al., 2004), or intergenic miRNA gene clusters (Altuvia et al., 2005). The biogenesis of miRNAs follows largely the canonical pathway which is introduced in a different review of this issue. For many enzymes of the miRNA pathway either the protein complex composition modulates activity for one particular, for families, or larger groups of miRNAs (most notably the microprocessor complex). Other steps in the miRNA biogenesis are also under tight control by miRNAs, protein products, or transcription factors. For more information in the area of miRNA regulation see another review in this issue or refer to recent reviews by Davis-Dusenbery and Hata (2010) as well as Newman and Hammond (2010).

Despite the great effort that has been put into the elucidation of the miRNA pathway, not much is known which would facilitate computational modeling that is based on clear processing facts instead of data mining approaches. In general hairpin structures are modeled and the parameters are used to distinguish true from false miRNA hairpins. This approach is complicated by the fact that a proper negative data set is not available.

Two computational ways to determine whether a sequence is a miRNA are currently employed. One of them is based on homology to known closely related miRNAs (evolutionary conservation). MiRscan (Lim et al., 2003), miRseeker (Lai et al., 2003), and PalGrade (Bentwich et al., 2005) are prominent examples for algorithms employing evolutionary conservation. This method is, however, impeded by the claim that miRNA evolution seems to progress at a high rate (Lu et al., 2008; Liang and Li, 2009). Furthermore, homology modeling rarely allows the detection of novel miRNAs but rather cements the current understanding of miRNAs (Bentwich et al., 2005) and it may, therefore, be advisable to focus on *ab initio* prediction. In the following we will therefore solely discuss how *ab initio* miRNA prediction can detect pre-miRNAs.

## MODELING THE BIOLOGICAL miRNA PROCESS

Relatively little is known about what constitutes a true miRNA but millions of hairpins can be found in a genome which makes the process of determining whether a hairpin is a miRNA difficult (Feng et al., 2011). A genome wide search for miRNAs would need to fold all parts of a genome, a problem which is computationally expensive and for which some algorithms have recently been compared (Janssen et al., 2011). Folding is necessary in order to generate hairpins that can then be evaluated for whether they contain a pre-miRNA that fits the applied model. As millions of putative pre-miRNAs can be generated from a genome, such as the human genome, it is essential to have highly accurate prediction algorithms. Current focus in this area is mostly the computational detection of pre-miRNAs. For the detection of pre-miRNAs, features are derived from the folded putative pre-miRNAs which discriminate between true and false miRNA hairpins. Machine learning algorithms are trained on known examples to discriminate between true and false pre-miRNAs.

In the following we will first comment on parameters that have been derived from miRNA hairpins, followed by a discussion of current algorithms for detection of pre-miRNAs and their accuracies. Afterward we ask the question whether in addition to the pre-miRNA detection the location of the mature miRNA sequence can also be predicted.

## WHAT CONSTITUTES A PRE-miRNA

All approaches for predicting miRNAs from genomic sequences depend on learning from examples since the underlying biological processes have not been completely elucidated. It is difficult to describe what exactly constitutes a proper pre-miRNA and how it differs from other hairpin structures. For this reason, more than 250 different parameters to describe a hairpin have been published in 12 studies performing *ab initio* pre-miRNA prediction (Lai et al., 2003; Pfeffer et al., 2005; Xue et al., 2005; Yousef et al., 2006; Jiang et al., 2007; Ng and Mishra, 2007; Bentwich, 2008; van der Burgt et al., 2009; Cakir and Allmer, 2010; Ding et al., 2010; Grundhoff, 2011; Ritchie et al., 2012). These parameters aim to describe features such as thermodynamic properties, sequence, and/or structure based, or probabilistic properties of a hairpin. **Table 1** shows the 10 most frequently used features in *ab initio* pre-miRNA prediction.

**Table 1:** **We analyzed all 12 studies which performed ab initio prediction of hairpins and selected the 10 most used features.** The most commonly used feature is the length of the loop of the hairpin, used in 6 out of the 12 studies.

Feature	Percent used
Hairpin loop length	50
Base pairing propensity	42
Minimum free energy probability	33
Minimum free energy of hairpin	33
Hairpin length	33
Percent of triple structure U((( in hairpin	33
Percent of triple structure U(.( in hairpin	33
Percent of triple structure C(.( in hairpin	33
Percent of triple structure A... in hairpin	33
Percent of triple structure G((( in hairpin	33

Features from the sequence based group are for instance single, di, and tri nucleotide counts and frequencies but also comparative features like the surplus of CG over AU as defined by van Ham and colleagues (van der Burgt et al., 2009). Parameters that describe structure include the hairpin loop length, number of bulges, and maximum bulge size among others. Sixteen hybrid features are introduced by Zhang and colleagues (Xue et al., 2005) which include both sequence information and structural information based on one central nucleotide and the bonding properties of the surrounding two nucleotides (see **Table 1**, row 6). Thermodynamic properties of a miRNA hairpin are for example its minimum free energy, its enthalpy, and its entropy; features which were used by for example in microPred (Batuwita and Palade, 2009) which is not a pure *ab initio* prediction tool but uses some evolutionary conservation information. Probabilistic features usually evaluate a feature of the other groups in respect to a set number of shuffled sequences to determine whether a pre-miRNA is a true miRNA hairpin. Van de Peer and colleagues introduced this analysis for minimum free energy (Bonnet et al., 2004). Whether it is beneficial to use such a transformed measure or use the minimum free energy calculation directly in machine learning is unclear, but not very likely.

Unfortunately, the predictive power of these features has not been analyzed in depth. Even despite their redundant usage their predictive quality has not been established which may be due to problems stemming from the absence of negative data. Another issue is the use of features which may be redundant or highly correlated so that they would lead to over estimation of some features, in turn leading to lowered prediction accuracy. One example can be the minimum free energy and the statistical transformation of the minimum free energy which are used in tandem in some studies (e.g., dG = mfe and zG in Ng and Mishra, 2007).

All 12 *ab initio* studies that attempt detection of miRNA hairpins have a unique combination of features. Some overlaps occur and some studies do not add new features but use a combination of previously described parameters. The features that are used to describe the miRNA hairpins are then used for learning the difference between true and false pre-miRNAs.

## MACHINE LEARNING FOR THE DETECTION OF PRE-miRNAs

Given the parameters that describe a pre-miRNAs, rules can be established from known examples that serve as training data in supervised learning.

### TRAINING DATA

For most machine learning approaches, which have been employed in pre-miRNA detection, it is necessary to have both positive and negative examples but in many problems in biology and especially for the prediction of pre-miRNAs, negative examples are hard to come by (Yousef et al., 2008; Ding et al., 2010; Wu et al., 2011; Ritchie et al., 2012). In order to generate negative data random sequences of similar length as the positive examples can be generated. Hairpins that occur in other RNA structures like tRNAs can be used, but there is no guarantee that these cannot act as miRNAs. Pseudo hairpins have been created (Ng and Mishra, 2007) and have been widely used. Negative examples can also be generated on the premise that a pre-miRNA does not contain another overlapping miRNA hairpin (Ambros et al., 2003). Positive data is readily available and most algorithms derive their positive examples from miRBase (Griffiths-Jones, 2010), but recent studies uncovered that caution is needed when deriving positive data from miRBase (Wang and Liu, 2011; Ritchie et al., 2012). Nonetheless, since positive examples are available and because negative examples are not one-class classifiers have been tried (Yousef et al., 2008).

### SUPERVISED LEARNING

Classification is a classic data mining discipline and many algorithms are available for supervised learning. From these algorithms naïve Bayes induction (Yousef et al., 2006), random forest (Jiang et al., 2007), and support vector machine (Pfeffer et al., 2005; Xue et al., 2005; Ng and Mishra, 2007; Ding et al., 2010; Ritchie et al., 2012) have been used. The basic strategy for supervised learning is to define positive and negative examples and some discriminating parameters to discriminate among the examples provided (see above). Although the machine learning algorithms employed may have some influence on the outcome of the prediction, we believe that the impact of proper test and training sets and well defined parameters are much higher. Therefore, the choice of supervised learning method seems to be negligible.

## OTHER APPROACHES

A strategy which does not employ machine learning for *ab initio* prediction of miRNAs is to determine the data distribution of selected parameters and then define a linear combination to describe a true hairpin (Bentwich, 2008), require thresholds that need to be passed (Cakir and Allmer, 2010), or define a likelihood (van der Burgt et al., 2009).

### PREDICTION ACCURACY

All studies which have reported new *ab initio* approaches to pre-miRNA prediction have used different data sets, which makes it impossible to compare the accuracy of these algorithms without rerunning them on the same data set. In addition to that, not all studies report prediction accuracy. Furthermore, some of the studies have different underlying aims which complicate a direct comparison even further. Lastly, there is no fully annotated available genome which would allow a proper accuracy assessment on real data. Therefore, the reported accuracies which will be very briefly recounted in the following are to be viewed as anecdotal.

Rubin and colleagues calculated their sensitivity in respect to the number of miRNAs they found, and which had already been described for *Drosophila melanogaster*. They detected 18 of 24 known miRNAs and reported a sensitivity of 75%, but did not offer specificity or accuracy measures (Lai et al., 2003). Zhang and colleagues trained a support vector machine to distinguish between real and pseudo human pre-miRNAs and achieved a sensitivity of 93% at a specificity of 88% (Xue et al., 2005). Margalit and colleagues (Altuvia et al., 2005) investigated viral miRNAs which can regulate host genes, using SVM classification, and report a sensitivity of 97% at a specificity of 71%. Showe and colleagues used naïve Bayes classification and reached a sensitivity of 97% at a specificity of 91% for mouse (Yousef et al., 2006). Lu and colleagues (Jiang et al., 2007) reused the same approach as Zhang and colleagues (Xue et al., 2005). Differently, they added a *P*-value and minimum free energy to the classification parameters and also used a different classification algorithm. They achieved a sensitivity of 95% at a specificity of 98%. MiRenSVM an algorithm combining three SVM classifiers achieved a sensitivity of 93% at a specificity of 97% (Ding et al., 2010).

We have recently assessed four studies in an attempt to independently establish the relative prediction accuracy of *ab inito* pre-miRNA prediction tools and found that even the best among these (accuracy: 0.986 on the pseudo hairpin data set from Ng and Mishra, 2007) would not be accurate enough to extract pre-miRNAs from the human genome with an error rate that would be acceptable to perform experimental validation for all predictions (Sacar and Allmer, manuscript in preparation). Assuming 11 million hairpins in the human genome (Bentwich, 2008) and an accuracy of 98.6% the number of potential false positive results would amount to 154000, a figure that is not acceptable when attempting experimental validation in the light of the fact that only a few thousand true miRNAs are expected (Berezikov et al., 2005).

A process even more difficult than the mere selection of whether a hairpin is a pre-miRNA is exactly locating the miRNA within the hairpin.

## WHERE IN THE HAIRPIN IS THE MATURE miRNA?

Hertel and Stadler (2006) claim that the mature miRNA may occur anywhere within the hairpin, but that is against experimental knowledge which established some rules for Drosha and Dicer cleavage (Zeng and Cullen, 2005; Han et al., 2006; MacRae et al., 2006; Zhang, 2010) which is likely due to their study predating many of these experimental findings. Their knowledge may stem from an analysis of miRBase which contains an abundance of dubious miRNAs which do not conform to some of the structural characteristics of miRNAs and are more likely other small RNAs with the same effect like siRNAs or piwiRNAs. Due to these problems, hand curated miRNA databases for miRNAs like Ssa miRNAs DB are now being developed (Reyes et al., 2012).

We tried to predict the location of the miRNA in the hairpin post-targeting by first taking the complete possible mature miRNA sequence and then narrowing it down based on BLAST (Altschul et al., 1990) results against 3′UTRs (Cakir and Allmer, 2010). Clearly, this approach, which we tried for *Toxoplasma gondii*, would not be scalable to the human genome and therefore other methods need to be explored.

Many programs have been developed for the detection of pre-miRNAs, however, only few of them are able to find the mature miRNA sequence within the hairpin (Gkirtzou et al., 2010; Xuan et al., 2011).

Huang and colleagues developed MaturePred which uses two-stage sample selection to predict the mature miRNAs for plants and animals (Xuan et al., 2011) based on a number of features which they compared between known miRNA:miRNA^⋆^ duplexes and pseudo ones. Some of the parameters they adopted are also used in pre-miRNA prediction algorithms and thus their method suffers likewise from missing negative data sets.

Poirazi and colleagues developed a method for localization of the mature miRNA within a pre-miRNA using parameterization and naïve Bayes classification (Gkirtzou et al., 2010). Among the features they used, some triplets and their relative position within the sequence turned out to be the most important qualifiers. They compared their software, MatureBayes, with BayesMiRNAfind (Yousef et al., 2006) and ProMiR (Nam et al., 2005), two tools with a different purpose than MatureBayes but which could potentially be used for the same purpose. They performed the comparisons in order to show that a naïve adaptation of non-specialized tools cannot outperform MatureBayes.

Tao (2007) employed thermodynamic and structural feature conservation among species to predict the location of the mature miRNA but in respect to the length of a mature miRNA the deviance of the predicted start site to the actual start site is quite large.

Ma and colleagues developed a hybrid experimental and computational approach which they used to determine the location of the mature miRNA for a small sample (Song et al., 2010).

Some progress has been made in the field and the approximate localization of the mature sequence seems to be in reach, but length variability and modifications to the mature miRNA are not accounted for by any of the proposed algorithms. These modifications have however a great impact on the viability or the target of a mature miRNA (Wang et al., 2011) and need to be considered in the future.

## CONCLUSION

Mature miRNAs are by no means independent of their processing pathway. It is essential that the processing steps from RNA polymerase to RNA-induced silencing complex (RISC) incorporation and silencing are performed to produce a mature miRNA. Therefore, it is impossible to separate the rules for generation of mature miRNA sequences from the underlying biological processes and they need to be modeled entirely for prediction of miRNAs.

Recently, a large number of additional regulatory options have become known and it has become clear that miRNAs can be regulated in many specific ways and in turn regulate in many specific ways, for example see Guil and Cáceres (2007).

It seems difficult to model all these specifics in computer algorithms as we are only beginning to understand the underlying biological pathway and its mode of regulation (Winter et al., 2009; Choudhuri, 2010).

Setting aside all the problems it is currently possible to find new miRNAs with a combination of experimental and computational research as was exemplified by Mowla and colleagues (Parsi et al., 2012) who used a variety of computational tools in concert to find a new putative miRNA in an intron of the NGFR gene which they then confirmed experimentally.

The field of computational prediction of miRNAs is nowhere near maturation yet tools are used and new ones are being developed. One of the benefits of using immature computational analysis strategies is that they often generate testable hypotheses and by that drive further research. This leads to concurrent synergistic increase in knowledge and in maturity of computational analysis tools.

## Conflict of Interest Statement

The authors declare that the research was conducted in the absence of any commercial or financial relationships that could be construed as a potential conflict of interest.
